# Engineering the Coordination Sphere of Isolated Active Sites to Explore the Intrinsic Activity in Single-Atom Catalysts

**DOI:** 10.1007/s40820-021-00668-6

**Published:** 2021-06-07

**Authors:** Xin Wu, Huabin Zhang, Shouwei Zuo, Juncai Dong, Yang Li, Jian Zhang, Yu Han

**Affiliations:** 1 State Key Laboratory of Structural Chemistry, Fujian Institute of Research on the Structure of Matter, Chinese Academy of Sciences, Fuzhou, 350002 People’s Republic of China; 2 KAUST Catalysis Center, Physical Sciences and Engineering Division, King Abdullah University of Science and Technology, Thuwal, 23955-6900 Saudi Arabia; 3 Beijing Synchrotron Radiation Facility, Institute of High Energy Physics, Chinese Academy of Sciences, Beijing, 100049 People’s Republic of China; 4 Advanced Membranes and Porous Materials Center, Physical Sciences and Engineering Division, King Abdullah University of Science and Technology, Thuwal, 23955-6900 Saudi Arabia

**Keywords:** Isolated atoms, Coordination sphere, Intrinsic activity, Single-atom catalysts

## Abstract

Reducing the dimensions of metallic nanoparticles to isolated, single atom has attracted considerable attention in heterogeneous catalysis, because it significantly improves atomic utilization and often leads to distinct catalytic performance. Through extensive research, it has been recognized that the local coordination environment of single atoms has an important influence on their electronic structures and catalytic behaviors. In this review, we summarize a series of representative systems of single-atom catalysts, discussing their preparation, characterization, and structure–property relationship, with an emphasis on the correlation between the coordination spheres of isolated reactive centers and their intrinsic catalytic activities. We also share our perspectives on the current challenges and future research promises in the development of single-atom catalysis. With this article, we aim to highlight the possibility of finely tuning the catalytic performances by engineering the coordination spheres of single-atom sites and provide new insights into the further development for this emerging research field.

## Highlights

- All the coordination engineering strategies, such as tuning the coordination species, the coordination number of the active centers, heteroatoms interactions within the support, synergetic interaction between neighboring metal monomers, and spatial microenvironment, have been summarized and discussed in detail.
- Various single-atom catalysts (SACs) with different coordination spheres in energy conversion driven by thermal, light and electric energy have been systematically reviewed.
- The current key challenges in SACs for energy conversion are pointed out, and some potential strategies/perspectives are proposed.

## Graphical abstract

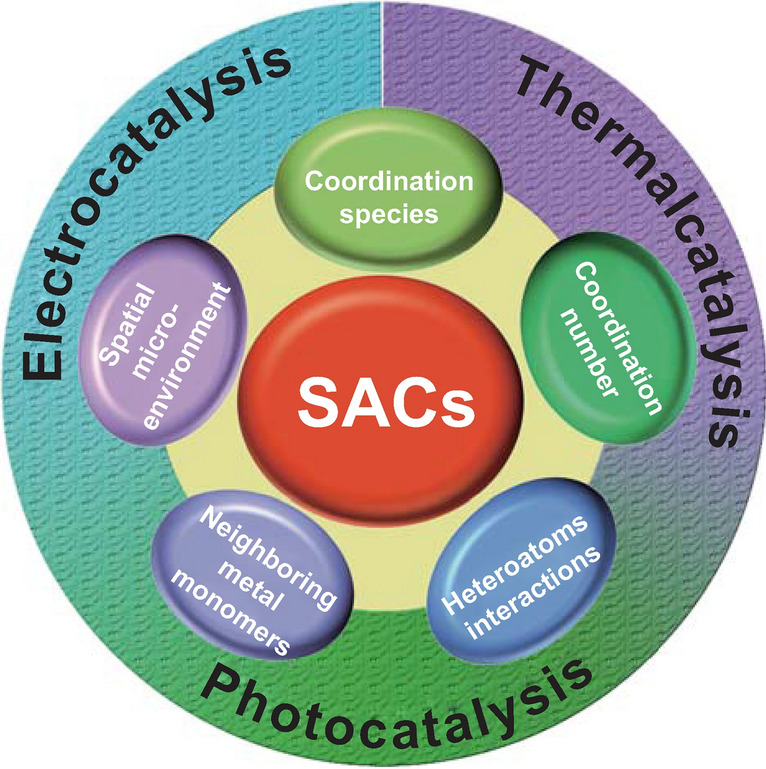

## Introduction

Single-atom catalysts (SACs) composed of isolated metal atoms as the active sites are considered to bridge the gaps between the homogeneous and heterogeneous catalysts [1–10]. The concept of “single-atom catalysts” was firstly proposed by Zhang and colleagues in 2011. In their pioneering work, they demonstrated that the isolated, single Pt atoms stabilized on an iron oxide support have extraordinary activity for CO oxidation [11]. Subsequent studies indicated that in SACs, the atomic dispersion of catalytic centers with nearly identical configuration not only represents the extreme atomic utilization, but also offers enormous opportunities to elucidate the underlying catalytic mechanism at atomic level [12–15]. However, preparing SACs with atomic precision is challenging, due to the high surface energy and tedious synthetic procedures, which largely hinders their practical applications. Thus it is of vital significance to develop synthetic strategy to prepare SACs with robust and well-defined structures from both academic and industrial points of view [16].

The reactive centers in SACs are greatly modulated by the surrounding atoms via the strong electronic coupling [17–22]. Thus, regulating the coordination sphere of the reactive center will significantly influence the intrinsic activity and stability of SACs, promoting their rapid optimization to satisfy the requirements of targeted reactions [23, 24]. Tremendous research endeavors have been devoted to tuning the performance of isolated reactive centers via coordination engineering [25–28]. At present, several instructive articles have reviewed the SACs fabrication and coordination environment regulation. Most works merely focus on the metal-support interactions and reaction mechanisms for electrochemical or photocatalytic reactions, lacking a comprehensive and systematical understanding of strategies toward promoting various catalytic reactions based on SACs [29–32]. Recently, the extensive exploration of various modulation strategies for SACs extends their application in wider catalysis systems. Therefore, a timely summary is highly needed on this fast developing area.

**Fig. 1:**
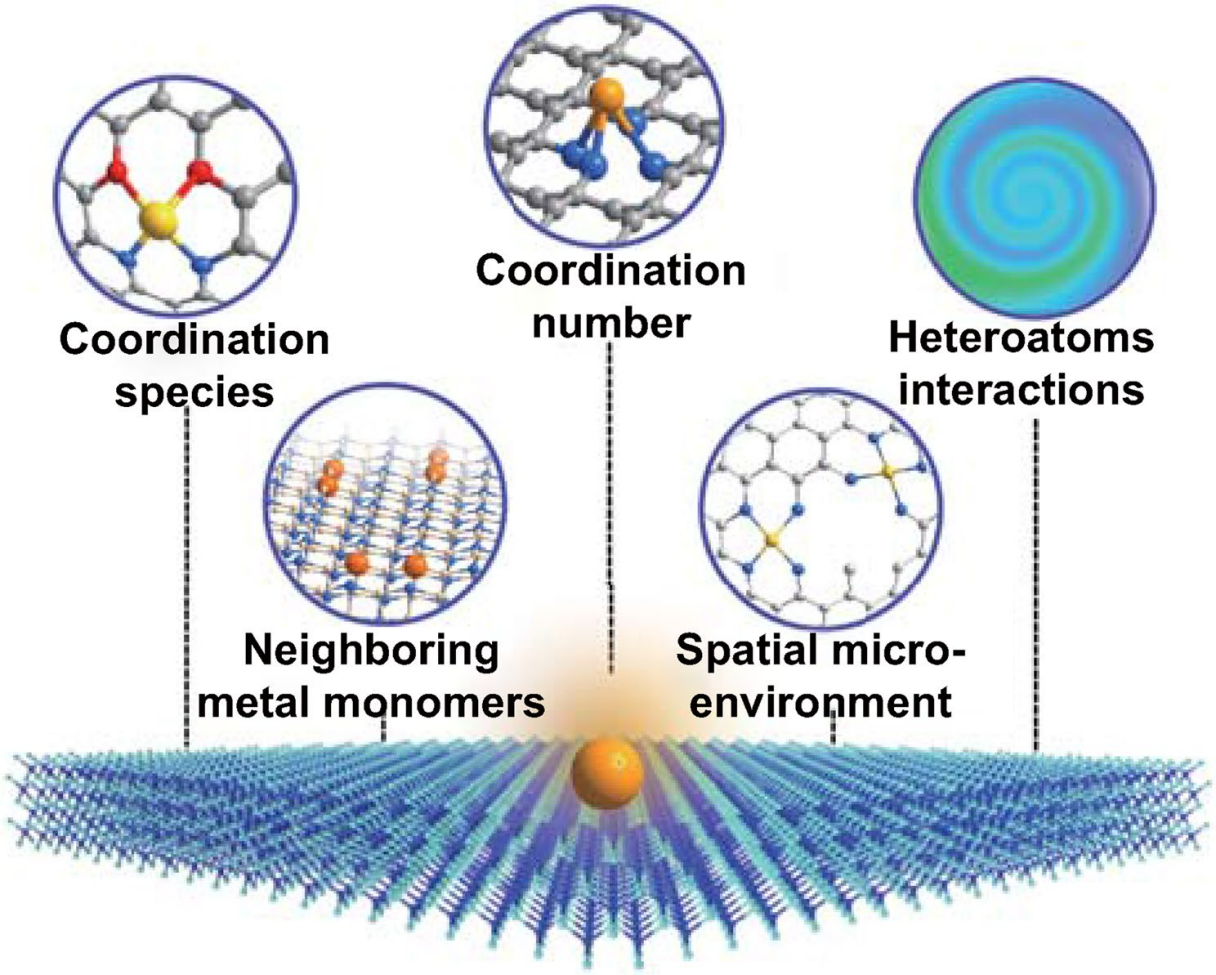
Schematic illustration of the strategies to engineer coordination sphere of isolated active sites

Herein, we summarize the latest advances in modulating the coordination spheres of SACs and analyze the key structural characteristics those influence the intrinsic activity of isolated reactive sites (Fig. 1). We center our discussions on the direct structure–performance relationship of the isolated reactive centers and the underlying mechanisms for the particular reaction types. Through some examples, we demonstrate how the catalytic selectivity, intrinsic activity and stability of SACs can be optimized by engineering the coordination sphere of the reactive centers. In the last section, we outline the current challenges and possible solutions associated with SACs. We expect that this review will inspire a more comprehensive understanding and in-depth discussion on the SACs-involved catalytic mechanisms, thus promoting the further development of this emerging research area.

## Rational Designs of SACs

The successful design of SACs requires the harmonization of multiple demands, such as high stability, uniform active sites, tunable electronic, geometric geometry and so on. However, it is still challenging to prevent the aggregation as the ultrahigh surface energy of isolated reactive centers. To address this critical issue, enormous attention has been focused on the stabilization and confinement of isolated metal atoms. In the current section, we will briefly introduce various strategies for producing SACs.

### Wet-chemistry Strategies

Generally, the wet-chemistry strategies include co-precipitation, impregnation [33, 34] and photochemical strategy, etc. In the wet-chemistry routes, consecutive processes are combined. The metal species are first anchored on various supports, followed by drying and annealing. At last, SACs with homogeneously dispersed reactive centers can be obtained after further reduction or activation. Although the SACs prepared by these strategies usually faced up with some drawbacks, such as low loading contents and poor dispersion of single atoms, wet-chemistry strategies have been extensively adopted as effective and convenient methods to prepare SACs [35, 36]. Zhang and colleagues have successfully developed a series of SACs via co-precipitation method with the metal atoms ranging from Pt, Ir to Au [11, 37–39]. When the aqueous solution of chloroplatinic acid and ferric nitrate is mixed with sodium carbonate at a finely tailored reaction temperature and pH value, Pt species can be anchored on the defects of FeO_x_. Calcination and reduction processes are further proceed to obtain the final Pt SACs.

**Fig. 2:**
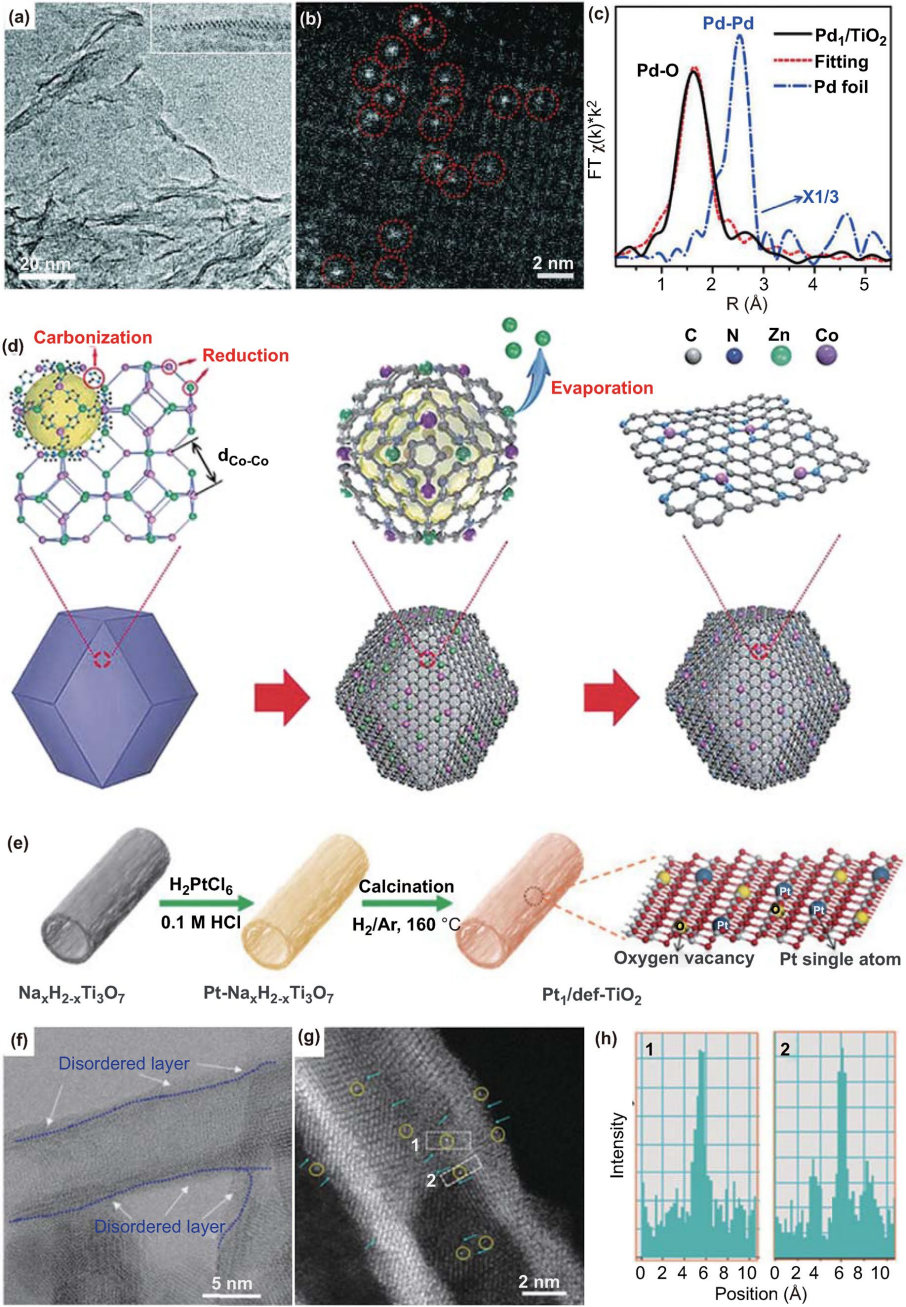
**a** Representative TEM image of Pd_1_/TiO_2_. The inset is an aberration-corrected STEM image for cross sections of ultrathin TiO_2_(B), showing that it is composed of only two layers of Ti atoms. **b** HAADF-STEM image of Pd_1_/TiO_2_. **c** FT-EXAFS spectra of Pd_1_/TiO_2_ and bulk palladium foil at the Pd K-edge, showing the surrounding atoms adjacent to Pd atoms. Reproduced from Ref. [41] with permission. Copyright 2016, American Association for the Advancement of Science. **d** The formation of Co SAs/N–C. Reproduced from Ref. [54] with permission. Copyright 2016 Wiley–VCH Verlag GmbH & Co. KGaA, Weinheim. **e** The preparation process of the Pt_1_/def-TiO_2_ catalyst. **f** HRTEM image and **g** AC HAADF-STEM image of the Pt_1_/def-TiO_2_ catalyst. The Pt atoms are marked by the yellow circles. The defective structure of the TiO_2_ support is marked by a blue arrow. **h** The intensity profiles obtained in regions 1 and 2 in **g**. **e–h** Reproduced from Ref. [61] with permission. Copyright 2020 Wiley–VCH Verlag GmbH & Co. KGaA, Weinheim

Ag alloyed Pd SACs supported on silica gel (AgPd/SiO_2_) have been prepared by a simple impregnation method. [40] After impregnation of silica gel with Pd(NO_3_)_2_ and AgNO_3_ solutions, bimetallic AgPd/SiO_2_ is obtained via calcination in air at 400 °C for 2 h. In the reduction process, Pd atoms are isolated on the optimal Pd/Ag surface ensembles. Zheng and coworkers have synthesized stable Pd/TiO_2_ SACs with the Pd loading content of 1.5 wt% through a photochemical method (Fig. 2a–c) [41]. Ethylene glycolate decorated ultrathin TiO_2_ nanosheet is selected as the support, which can generate radicals under ultraviolet light to promote the removal of Cl^−^ ligand from Pd sites in H_2_PdCl_4_ and form the isolated Pd atoms via Pd–O bonds. The finely synthesized Pd_1_/TiO_2_ catalyst shows prominently high stability and activity in the hydrogenation of C=C and C=O.

### Metal–organic Frameworks (MOFs)-Participated Approaches

MOFs as functional porous materials have attracted enormous attention for many applications related to catalysis, gas adsorption and separation [42–46]. The diverse architectures and compositions make it possible to incorporate multifunctional catalytic centers in MOFs and tailor the configuration of SACs, which render them to be unsurpassed precursors for the generation of SACs [47–49]. More importantly, mechanistic insights into catalytic activity origin of SACs can be achieved through the precise control of the structures of SACs.

The MOF itself can be applied as the support for stabilizing the isolated reactive centers. Ye and colleagues have realized the dispersion of isolated Co active centers into the porphyrin unit of the MOF-525 [50]. The porphyrin units not only work as the anchoring site for isolating the reactive centers, but also can modularize the coordination geometry of the isolated Co centers. Directional transfer of photo-generated electrons from porphyrin units to the isolated Co centers has been observed, realizing the supply of photo-generated electrons for the CO_2_ reduction over the isolated Co centers. Lin and colleagues have further applied MOFs to stabilize the low coordinated Ir species for methane borylation [51]. The isolated Ir atoms in the mono(phosphine)-Ir-based MOF (Zr-P_1_-Ir) adopt the distorted tetrahedral geometry and are coordinated by one chloride, one bidentate 1,5-cyclooctadiene, and one mono(phosphine) from the MOF ligand. Comparing with some other Ir catalysts, Zr-P_1_-Ir delivers more impressive catalytic performance in methane borylation to exclusively afford CH_3_Bpin with a turnover number (TON) of 127 at 110 °C. Furthermore, by utilizing the post-synthetic modification of MOFs, individual metal atoms can be anchored and maintained in high dispersion [52]. For instance, isolated Pt atoms have been firmly trapped by the four pyrrolic nitrogen sites of porphyrin centers in MOFs through Pt(II) metalation and a followed reduction process [53].

Besides, the MOFs are widely used as the precursors for preparing SACs with a particular configuration. When bimetallic Zn/Co MOF is selected as a precursor for calcination, isolated Co atoms decorated porous carbon matrix can be constructed with the high loading content over 4 wt% [54]. In this process, Co nodes can be in situ-converted into isolated Co atoms by carbonization of the organic linker. The intentional mixed Zn atoms are volatile and can be easily removed at high temperatures, which can serve as a “fence” to avoid the generation of Co–Co bonds. The final single Co catalyst exhibits pronounced oxygen reduction reaction (ORR) activity with a more positive half-wave potential (0.881 V) than that of commercial Pt/C (Fig. 2d). Taking advantage of the uniformly distributed pores in MOFs, isolated metal atoms can be confined in the MOFs matrix [55]. Li et al. have reported the host–guest strategy to adsorb FeCl_3_ molecules within the confined space of Zn/Co bimetallic MOFs [56]. Fe ions can be confined in the graphitization process and coordinated with adjacent Co atoms, achieving an elegant hybrid with porphyrin-like Fe-Co dual sites embedded carbon matrix.

### Defect Immobilized Approaches

Defects exist widely in nanomaterials, such as step edges, caves and intrinsic defects, which are beneficial to immobilize isolated metal atoms and alter the geometric and electronic properties for improving the reaction activity [57, 58].

Step edges are the most ubiquitous defects on the solid surfaces. Edges in the ceria have been applied for segregating the Pt atoms in the planar PtO_4_-configuration [59]. Experiments and density functional theory (DFT) results confirm the preferential segregation of Pt atoms at the steps with direct Pt-O bonding. The density of the step edges can be well tuned and thus afford the SACs with varied Pt loading amounts. Yan and coworkers have taken advantage of the fourfold caves in the phosphomolybdic acid for stabilizing the isolated Pt sites in high loading content [60]. The isolated Pt reactive centers are coordinated by four oxygen atoms in a distorted square-planar geometry. The developed catalyst exhibits impressive activity in hydrogenation of nitrobenzene and cyclohexanone. The intrinsic defects not only can be applied as the anchor sites, but also perform as the media for modulating the intrinsic catalytic activity of SACs. Li et al. have decorated the isolated Pt atoms on the intrinsic defects of the TiO_2_ support (Pt_1_/def-TiO_2_) as a superb photocatalyst (Fig. 2e–h) [61]. Notably, isolated Pt atoms can promote the adjacent TiO_2_ moieties to produce surface oxygen vacancies and Ti^3+^ defects, giving rise to an exceptional photocatalytic performance for hydrogen evolution.

### Other Synthesis Methods

In the synthesis progress of SACs, some other approaches have also been well established. In this section, a brief introduction of other synthesis methods is provided, including atomic layer deposition (ALD), ball-milling, and high-temperature atom trapping. It should be noted that these methods typically involve expensive equipment and afford low yields, which are not favorable for large-scale production.

ALD is one of the high-vacuum physical deposition techniques, which involves the self-limiting binary reactions between the substrate and gaseous precursor and allows precisely controlled deposition of diverse structures on various substrates. In the ALD process, the chosen substrate is alternately exposed to various vapor precursors. The metal is deposited in an atomic layer-by-layer manner. Sun et al. have stabilized the isolated Pt atoms on the surface of graphene nanosheet via ALD technique [62]. When the support is exposed to (methylcyclopentadienyl)-trimethylplatinum (MeCpPtMe_3_), the limited supply of oxygen on the graphene nanosheets disenables the oxidization of full ligands, generating Pt species in monolayer. Subsequently, the new oxygen layer can be formed on the Pt with further exposure to the oxygen, which can continue to adsorb Pt in a layer-by-layer manner. The deposition of Pt can be precisely controlled by adjusting the cycle numbers of ALD. These single Pt atom catalysts exhibit much higher activity in the methanol oxidation reaction and CO adsorption. However, this route is limited by the high cost and rigorous manipulation. The catalysts obtained via ALD approach usually possess non-uniform size and shape, which is not favorable for deeply understanding the correlation between local structures and reaction activities.

High-energy ball milling approach enables the breaking and reconstruction of chemical bonds in molecules and thus shows great potential to synthesize SACs. Bao and colleagues have reported the preparation of graphene confined metal-N_x_ catalysts via the ball milling approach for the first time [63–65]. In the synthesis process, graphene nanosheets (GNs) are firstly obtained by ball milling the natural graphite powder, then iron phthalocyanine (FePc) is combined with the GNs for further ball milling. Due to the high energy input by the ball milling, FePc can be reconstructed, affording the FeN_4_ embedded graphene.

The SACs can also be constructed through the thermal transformation of bulk particles into isolated reactive centers. Datye et al. have employed ceria powders with different exposed surface facets as supports to trap the individual platinum atoms [66]. When Pt particles deposited Al_2_O_3_ (Pt/La-Al_2_O_3_) mixing with ceria powders, Pt species can be emitted under high-temperature treatment and then migrate to the surface of CeO_2_ spontaneously. Various characterizations show that ceria polyhedrons and nanorods behave more effectively in avoiding the aggregation of Pt when comparing with the ceria cubes. Li and coworkers have further developed the thermal transformation strategy for converting the nanoparticles of precious metal (M = Pt, Pd, and Au) to isolated reactive centers in the inert atmosphere [67]. The mobile precious metal atoms are stabilized by the defects of nitrogen-doped carbon in the M-N_4_ geometry, providing driven force for the conversion of the nanoparticle to the isolated atoms.

## Coordination Engineering to Explore the Intrinsic Activity

Evaluating the contribution of the particular active site is an ideal approach to understand the origin of the catalytic process. SACs with the well-controlled configuration and homogeneous dispersion provide us an elegant platform for identifying the contribution of the isolated active sites and recognizing the key parameters for influencing the catalytic performance [60, 68, 69]. Thus, modulating the coordination spheres of SACs is an effective strategy to further explore the intrinsic activity of the catalysts.

### Tuning the Coordination Species of Active Centers

**Fig. 3:**
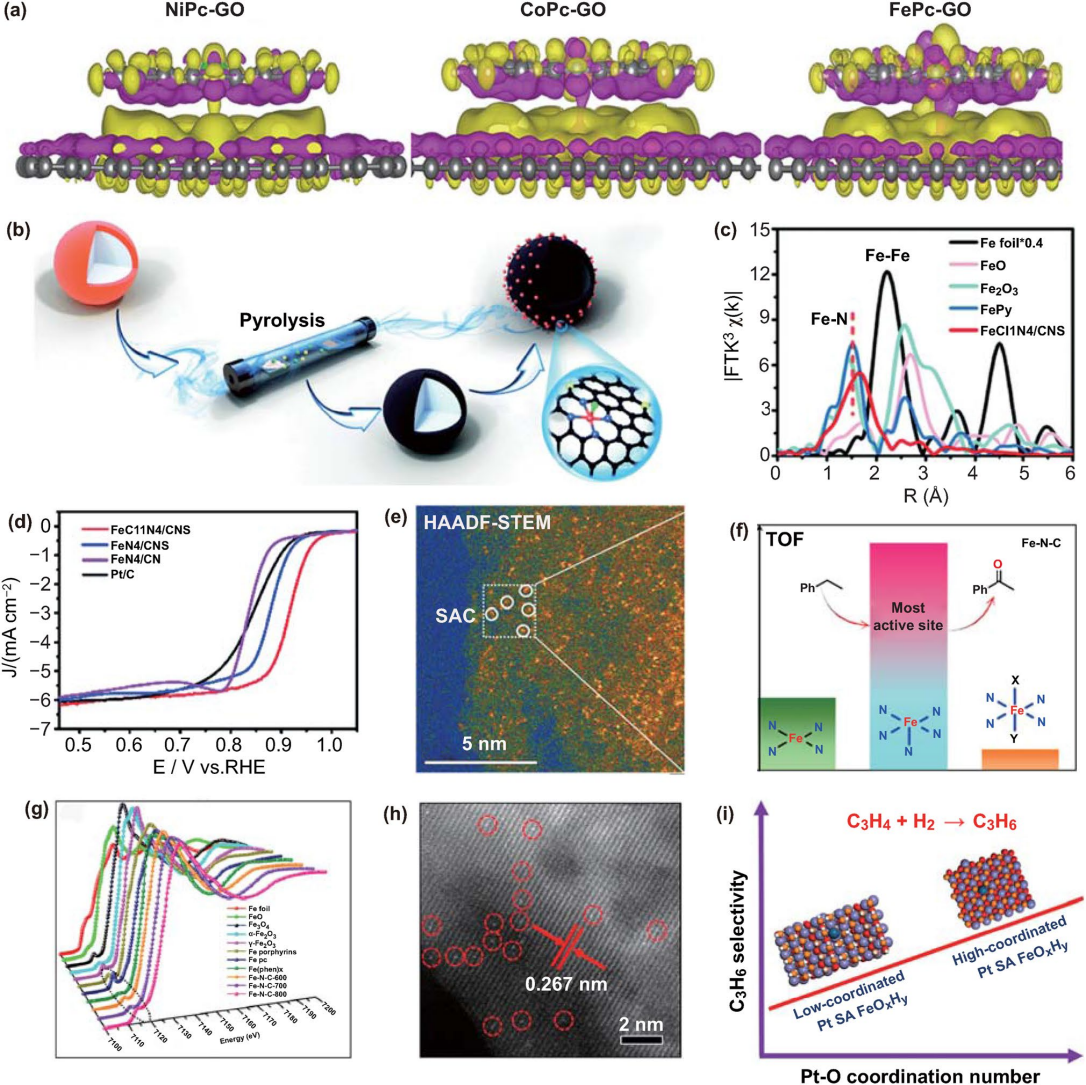
**a** DFT simulations of the synergistic effect between MPcs (M = Ni, Co, Fe) and GO. Isosurface plots of charge redistribution for NiPc-GO, CoPc-GO and FePc-GO. Yellow or violet color represents the accumulation or depletion of electrons, respectively. Reproduced from Ref. [70] with permission. Copyright 2020 American Chemical Society. **b** Schematic illustration of the synthesis of FeCl_1_N_4_/CNS. **c** Fourier transform (FT) at the Fe K-edge of the FeCl_1_N_4_/CNS, FePy, FeO, Fe_2_O_3_ sample and Fe foil. **d** ORR polarization curves in O_2_-saturated 0.1 M KOH. **b–d** Reproduced from Ref. [72] with permission. Copyright 2018 The Royal Society of Chemistry. **e** HAADF-STEM images of Fe–N–C. **f** Fe^III^N_5_ affords the highest turnover frequency for the selective oxidation of the C–H bond. **g** The normalized XANES spectra at the Fe K-edge of different samples. The dotted ellipse shows the pre-edge peak at 7117 eV. **e–g** Reproduced from Ref. [73] with permission. Copyright 2017 American Chemical Society. **h** High-resolution AC-STEM image of isolated Pt atoms. **i** The propylene selectivity increases as the coordination number of Pt–O increases. **h-i** Reproduced from Ref. [76] with permission. Copyright 2020 American Chemical Society

It is noteworthy that the active centers with nearly identical coordination sphere, but varied coordination species deliver distinct catalytic activities. The coordination species is a quite crucial parameter for influencing the catalytic performance. Patzke and colleagues have confirmed the important role of metal species in tuning the electrocatalytic performance for ORR (Fig. 3a) [70]. They have developed a soft-landing molecular approach to decorate metal phthalocyanines with varied metal species (M = Co, Ni, and Fe) onto the graphene oxide layers, and successfully synthesized the well-defined SACs. Experimental results and DFT calculations suggest that the Fe species exhibits stronger affinity toward O_2_ species when comparing with other metal species, resulting in greatly accelerated reaction kinetics for the oxygen reduction.

Besides, introducing the exotic non-metal species into the coordination sphere is also an effective approach for adjusting the electronic structure of the reactive center. Zhang and co-workers have successfully introduced the N species into the first coordination sphere of the isolated Ni atoms [71]. Comparing with the Ni-C dominated coordination sphere, the Ni–N coordination with effective electron coupling can move down the Fermi level of the hybrid, resulting in greatly reduced adsorption energy of intermediates and highly accelerated reaction kinetics for oxygen evolution.

Li and colleagues have further introduced chlorine atoms into the coordination sphere of the isolated Fe centers, and constructed an elegant catalyst (FeCl_1_N_4_/CNS) for electrochemical ORR (Fig. 3b–d) [72]. Comparing with the catalyst FeN_4_/CNS without chlorine modification, the catalyst FeCl_1_N_4_/CNS delivers greatly improved catalytic activity with a half-wave potential of 0.921 V. Calculation results reveal that the exceptionally outstanding ORR activity can be ascribed to the electronic coupling between the Fe reactive centers and coordinated chlorine atoms, resulting in heavily modified electronic structures and highly accelerated reaction kinetics.

### Controlling the Coordination Number of the Active Center

Given that the isolated reactive centers in SACs are stabilized by direct ionic and covalent coordination with the supports, the coordination number of the active sites should be an effective and reasonable descriptor for modulating the intrinsic activity of the SACs. Zhang and coworkers have successfully fabricated the exclusively dispersed Fe catalysts with different coordination numbers (FeN_x_, x = 4–6) (Fig. 3e–g) [73]. Combinatorial investigations demonstrate that Fe^III^N_5_ delivers the highest catalytic performance for selective oxidation of C-H bond, which is one order magnitude and three times higher than those of Fe^III^N_6_ and Fe^II^N_4_, respectively. The low activity of Fe^III^N_6_ should be ascribed to its saturated coordination between central Fe and surrounding N atoms, and no coordination space is left for reacting with the reactant. Zhao and colleagues have further decorated the isolated tungsten atoms onto the nitrogen-doped carbon nanosheets with precisely controlled W–N coordination numbers [74]. Experimental results clearly demonstrate that the isolated W atom coordinated by five nitrogen atoms exhibits remarkably enhanced ORR catalytic performance relative to those hybrids with W–N coordination number of 3 and 4. The coordination number-sensitive ORR performance of the single tungsten atom catalyst should be originated from the interaction between OH^−^ and the isolated W atoms moderated by the coordination number.

Coordination chemistry of the isolated reactive centers supported on metal oxide has also been explored with obvious progress. Parkinson et al. have decorated isolated Ir atoms into the lattice of Fe_3_O_4_ with varied geometries and applied the as-synthesized hybrids for CO adsorption [75]. Both the fivefold Ir atoms with octahedral coordination sphere and twofold Ir atoms with square-planar configuration exhibit greatly enhanced CO adsorption ability when comparing with the metallic Ir. Experimental results and DFT simulation clearly demonstrate the coordination unsaturated Ir atoms with coordination vacancies can effectively reactive with the CO molecules and thus boost the adsorption capacity.

Zhang and colleagues have further demonstrated that varying the coordination number of the isolated Pt centers can obviously improve the propylene selectivity during propyne semi-hydrogenation (Fig. 3h, i) [76]. They have modulated the coordination number of the isolated Pt centers from 3.43 to 5.04 over FeOOH supports by the thermal treatment. Experimental results and DFT simulations verify that the Pt SACs with reduced Pt-O coordination number is beneficial for propyne over-hydrogenation, resulting in greatly enhanced catalytic activity.

### Heteroatoms Interactions within the Support

**Fig. 4:**
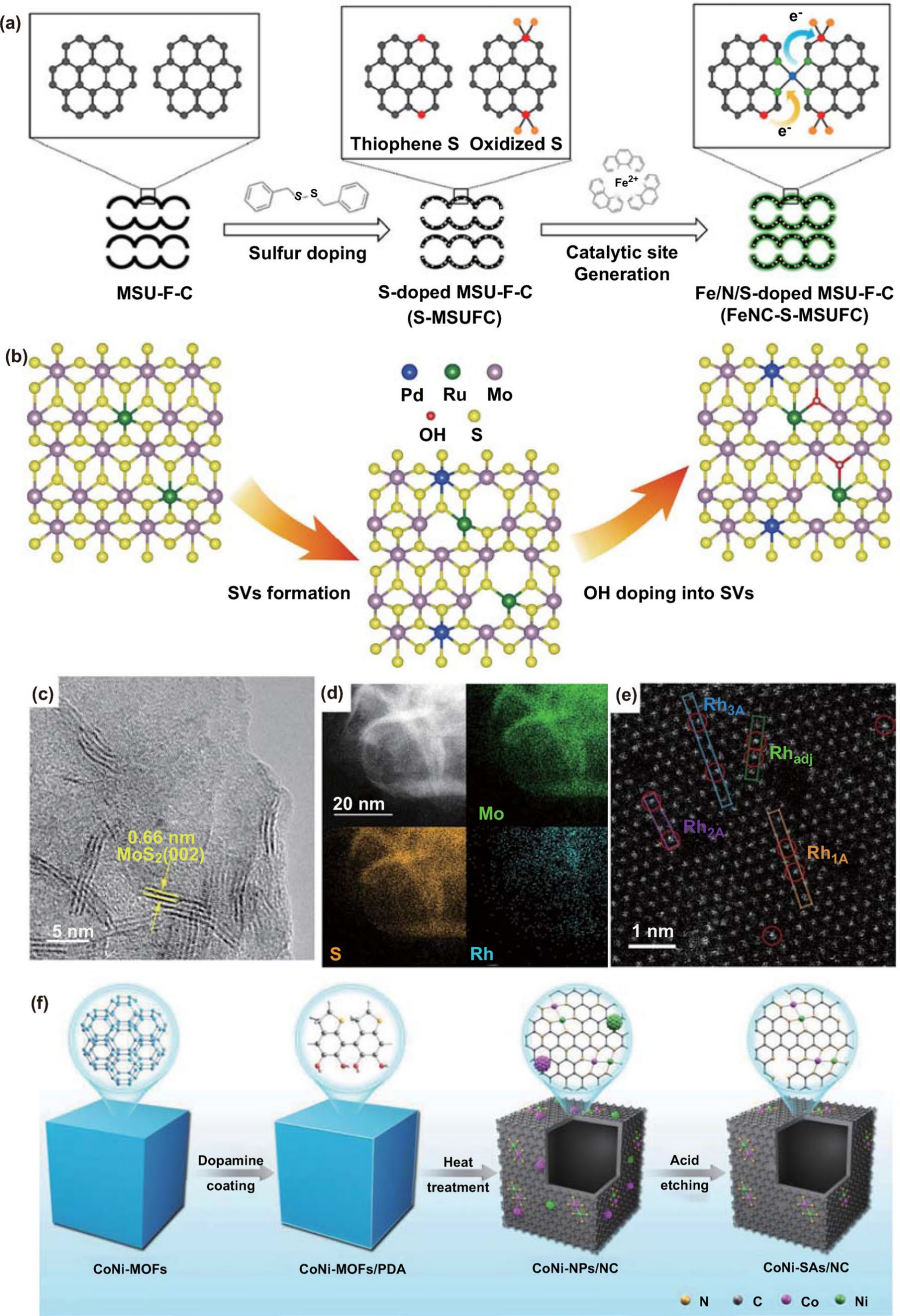
**a** Schematic representation of the synthesis of Fe/N/C catalysts with S-doped MSU-F–C. In the figure, the black, light green, blue, red, and orange spheres represent carbon, nitrogen, iron, sulfur, and oxygen atoms, respectively. Reproduced from Ref. [78] with permission. Copyright 2019 American Chemical Society. **b** Schematics for the synthesis strategy of Pd, Ru dual-doped MoS_2–*x*_OH_y_ phase. Blue, yellow, purple, green and red spheres represent Pd, S, Mo, Ru, and O atoms, respectively. Reproduced from Ref. [80] with permission. Copyright 2020 Springer Nature. **c** HRTEM image and **d** HAADF-STEM image with EDS mapping of the Rh-MoS_2_ sample. **e** Atomic-resolution HAADF-STEM image of the Rh-MoS_2_ sample. Rh_adj_ denotes the distribution of confined Rh atoms adjacent to each other. Rh_nA_ denotes the distribution of confined Rh atoms separated by n Mo atoms along the armchair direction. **c–e** Reproduced from Ref. [84] with permission. Copyright 2020 Wiley–VCH Verlag GmbH & Co. KGaA, Weinheim. **f** Schematic illustration of the synthetic strategy of CoNi-SAs/NC. Reproduced from Ref. [87] with permission. Copyright 2019 Wiley–VCH Verlag GmbH & Co. KGaA, Weinheim

Heteroatoms doping within the support has been recently developed as a simple and effective strategy to modulate the electronic structure of the isolated reactive centers. Guo and co-workers have synthesized a sulfur-doped electrocatalyst (Fe/SNC) with high density of atomically dispersed Fe sites [77]. The incorporated sulfur gives rise to a thiophene-like structure (C-S-C) in Fe/SNC. The localized electron density around Fe atoms can be reduced by the C-S-C moieties, leading to improved interaction with oxygenated species and boosted ORR activity. Lee and coworkers have decorated electron-withdrawing oxidized S group into the carbon plane and applied the as-synthesized hybrids for ORR (Fig. 4a) [78]. The electron-withdrawing effect can lower the d-orbital energy level of the isolated Fe atoms and thus boost the ORR activities. In contrast, the thiophene-like S functionalities behaving as electron donator to the carbon plane generate stronger adsorption of the ORR intermediates on the Fe-N_4_ sites and reduce the specific activity of ORR.

Li and co-workers have further modulated the electronic states of the active centers via decorating the phosphorus and sulfur species into the carbon matrix [79]. Experimental results and theory simulation suggest that the effective electron coupling between the isolated Fe atoms and surrounding S and P atoms should be responsible for the high-efficiency and satisfactory kinetics of ORR. Ge et al. have built a di-anionic surface on metal doped MoS_2_, in which sulfur anions can be molecularly substituted by –OH (Fig. 4b) [80]. The –OH group endows the interface with reactant dragging functionality. The well-conditioned surface, in conjunction with activated sulfur atoms by heteroatom metal doping as active sites, results in the greatly boosted hydrogen evolution reaction (HER) kinetics. The compositional advantage is also proved by introducing other metal species. The bimetallic catalyst of isolated Zn and Co atoms confined in sulfur-modified carbon architecture (ZnCo/NSC) shows an impressive ORR performance with the half-wave potential of 0.893 V, which outperforms the catalysts with only Zn or Co metallic sites [81]. Investigations demonstrate that interaction between the bimetallic sites can activate the O–O bonding and reduce the reaction barriers in the ORR process.

### Synergetic Interaction between Neighboring Metal Monomers

As the density of isolated active centers increases, the distance between two metal monomers can be greatly shortened and neighboring metal monomers are formed. The interaction between a pair of active monomers can generate a more optimized electronic structure and thus influence the related catalytic performance. Zeng and coworkers have demonstrated that pairs of Pt monomer stabilized by MoS_2_ can work in synergy to enhance the CO_2_ hydrogenation catalytic performance and undergo different reaction paths relative to isolated Pt monomer [82]. Formic acid and methanol are obtained in the hydrogenation of CO_2_ catalyzed by neighboring Pt monomers. In the absence of ensembles, isolated Pt atoms catalyze CO_2_ into methanol instead of the formic acid intermediates. Li et al. have synthesized the diatomic Fe_2_ clusters supported on g-C_3_N_4_, which exhibit outstanding catalytic performance for the alkene epoxidation [83]. The unique reactivity is benefited from the generated active oxygen species from the diatomic Fe_2_ clusters. In contrast, the isolated Fe atom catalyst is nearly inert. Deng et al. have realized an impressive HER activity by adjusting the distance between the isolated Rh atoms on MoS_2_ (Fig. 4c–e) [84]. DFT simulation further suggests that the optimized distance can trigger the activity of neighboring S atoms for hydrogen evolution, thus resulting in the maximized activity on the activated S atoms in the hybrid.

Employing two different neighboring monomers can provide a new reaction path to boost the catalytic selectivity and activity. Guo and coworkers have reported the neighboring Pt-Ru monomers stabilized by the N vacancy on g-C_3_N_4_ [85]. The neighboring Pt-Ru monomers with rich electrons are expected to own a higher activity than the Ru–Ru/Pt–Pt monomers or single Ru/Pt atom in the CO oxidation reaction. Mechanism investigations suggest that Pt-Ru pairs enable the formation of bridge-type O_2_ adsorption and thus optimize the O_2_ activation process. Sun and coworkers have fabricated discrete Zn-Co dual atomic centers on nitrogen-doped carbon to offer more concentrated active sites (Zn/CoN-C) [86]. Structural information and DFT calculations reveal that electronic structure can be modulated due to the formation of ZnCoN_6_ site, elongating the O–O bond length and accelerating the breaking of O_2_. The as-synthesized Zn/CoN-C catalyst delivers impressive ORR activities in both acid and alkaline media with a half-wave potential of 0.796 and 0.861 V, respectively. Hu et al. have synthesized binary Co–Ni sites anchored by N-doped hollow carbon nanocubes (CoNi-SAs/NC) (Fig. 4f) [87]. The synergistic effect of diatomic Co–Ni sites in porous carbon matrix endows CoNi-SAs/NC with optimized oxygen adsorption/desorption properties and decreases the reaction barriers for the oxygen involved catalysis, accelerating the bifunctional oxygen reduction/evolution reaction kinetics, surpassing other counterpart catalysts.

### Spatial Microenvironment

**Fig. 5:**
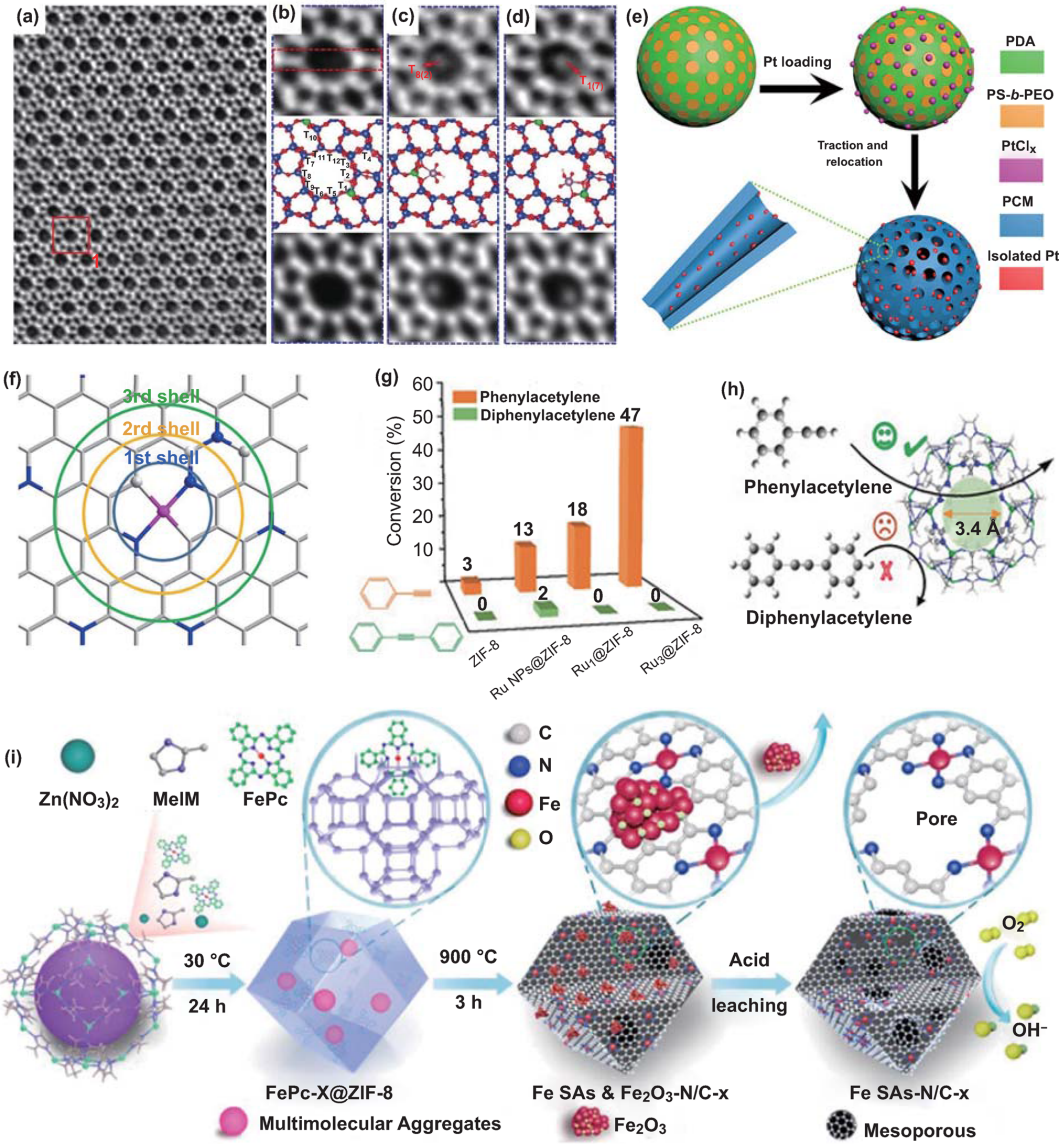
**a** A representative iDPC-STEM image of Mo/ZSM-5, showing the presence of off-center contrast in many 10-MR channels. **b–d** Zoomed-in areas of **a**, corresponding to three scenarios: empty channel **b**, and channels containing a MoO_3_H cluster bound at the T8 site **c** and at the T1 site **d**. Each panel includes the raw image (top), the calculated structural model (middle), and the simulated projected electrostatic potential (bottom). Si blue, O red, Al green, Mo pink, H white. **a-d** Reproduced from Ref. [88] with permission. Copyright 2020 Wiley–VCH Verlag GmbH & Co. KGaA, Weinheim. **e** Schematic illustration of the synthetic procedure of Pt@PCM. **f** Schematic description for the coordination shells for the isolated Pt over the graphene. **e–f** Reproduced from Ref. [89] with permission. Copyright 2018, American Association for the Advancement of Science. **g** Conversion for hydrogenation of phenylacetylene over Ru_3_@ZIF-8, Ru_1_@ZIF-8 and references. **h** Molecular sieving size-selectivity sketch of phenylacetylene and diphenylacetylene. **g–h** Reproduced from Ref. [93] with permission. Copyright 2019 Wiley–VCH Verlag GmbH & Co. KGaA, Weinheim. **i** Schematic illustration of Fe-N_4_ sites anchored on 3D hierarchically porous carbon. Reproduced from Ref. [94] with permission. Copyright 2018 American Chemical Society

Spatial confinement has been established as an effective strategy to develop SACs with impressive activity, selectivity and stability. Han and colleagues have realized the confinement of the isolated Mo atoms in the porous ZSM-5 matrix [88]. The integrated differential phase-contrast scanning transmission electron microscopy directly confirms the atomic dispersion of Mo species in the pores of the ZSM-5 support (Fig. 5a–d). Moreover, the specific Mo-Al interaction provides an opportunity for relocating the Al atoms in the framework of the ZSM-5. Lou and coworkers have developed an effective strategy for confining the isolated Pt atoms into the porous carbon matrix, which exhibits greatly boosted mass activity for electrocatalytic HER comparing with commercial Pt/C catalyst (Fig. 5e, f) [89]. Qiu and coworkers have decorated the isolated Pd atoms into the pores of MIL-101 (Cr), which delivers much higher hydrogenation activity than that of Pd adsorbed on the MIL-101 surface [90]. The authors ascribe the superior catalytic activity to the highly exposed Pd atom and confinement effect offered by the pores of MOFs.

Decorating the isolated metal species into the regular pores of the support can integrate their respective merits for catalysis. Hyeon et al. have confirmed the important role of the porous N-doped carbon structure of Fe–N–C catalysts for electrochemical ORR activity [91]. With the existence of mesopores, a large part of the physical surface area becomes reachable for the reactants and thus facilitates the kinetic accessibility of the active sites during the ORR. Moreover, porous zeolites and MOFs can serve as novel molecular sieves. Yu and colleagues have employed the oxygen atoms of MFI-type zeolites to stabilize isolated Ru atoms [92]. The zeolite channels facilitate shape selectivity in a manner that allows nitrobenzene with a smaller size to reach Ru sites while the bulkier 3-nitrotoluene is hindered by the zeolite pores. Similarly, the ZIF-8 pore structure allows contact of the reactant with the anchored Ru sites with absolute region-selectivity of catalyzing terminal alkynes but not internal alkynes (Fig. 5g, h) [93].

Defects are commonly existed in the spatial microenvironment and may cast some positive effects on the catalytic performances. Controlled construction of surface defects on supports has also been proven effective for developing SACs with well-defined microenvironments. The isolated reactive centers locating at the corner and edge sites always display a superior activity compared with the reactive centers confined in the bulk phase. Inspired by this, researchers set out to utilize highly curved supports to trap single metal sites. For example, edge-hosted Fe-N_4_ moieties have been prepared in hierarchically micro-mesoporous carbon matrix (Fig. 5i), which exhibit greatly improved ORR activity compared with those of intact atomic configuration [94]. Both experiments and DFT calculations reveal that such a defective Fe-N_4_ single site is a key to tailor the bonding structures of N and lower the overall ORR barriers. Song et al. have used onion-like carbon, instead of two-dimensional graphene, to support single Pt atoms and achieved the outstanding HER performance [95]. The working mechanism is uncovered by DFT results. Compared to the conventional catalysts, onion-like carbon with curve nature enables the accumulation of electrons around the Pt regions. The strong local electric field accelerates charge delivery and optimizes catalytic kinetics for HER. Lou et al. have implanted single Ru atoms with precise configuration into edge-rich carbon (ECM@Ru) for the electrocatalytic hydrogen evolution [96]. Comparing with the Ru-decorated carbon matrix (CM@Ru) particles without edges, ECM@Ru displays a 4.1 times higher mass activity at the overpotential of 100 mV. Experimental and calculation results reveal the key role of carbon edges in enhancing the local electric field around Ru monomers and accelerating the HER kinetics. They have also dispersed Ru atoms on multi-edged TiO_2_ spheres for photocatalytic hydrogen evolution [97]. The multi-edge TiO_2_ architecture greatly facilitates the charge separation and transport in photocatalysis process, further demonstrating the important role of the defects and edges in accelerating the reaction kinetics.

## Catalytic Applications of the Isolated Reactive Centers

Developing prominently stable, efficient and low-cost catalysts is crucial for the practical applications in energy conversion systems. Advanced SACs have emerged as promising paradigms with excellent activity, selectivity and stability. In the current section, we select several recent successful cases to show the potential and capability of SACs in energy conversion driven by thermal, light and electric energy.

### Thermal Catalytic Reactions

#### CO Oxidation

**Fig. 6:**
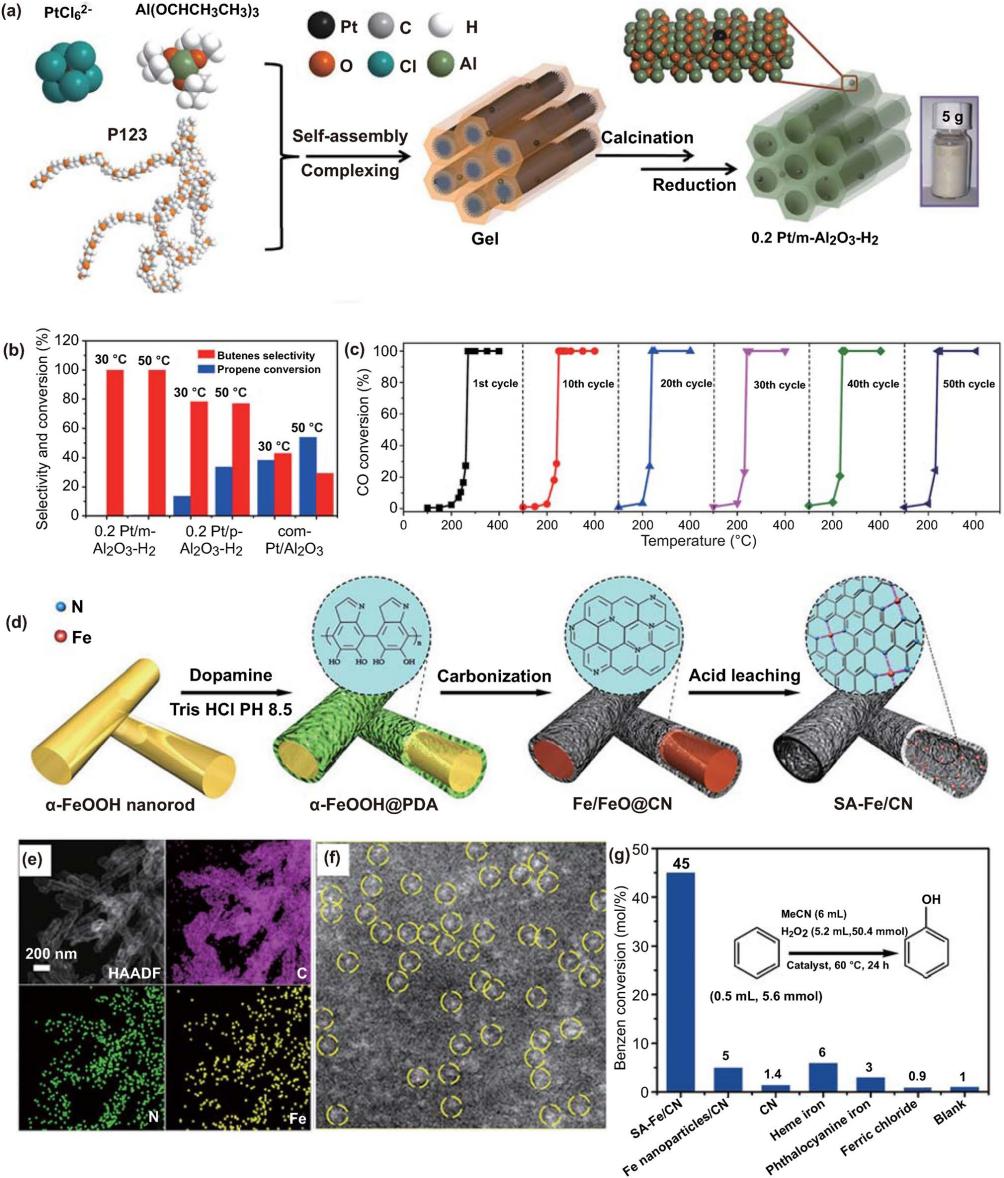
**a** Schematic illustration of the 0.2Pt/m-Al_2_O_3_-H_2_ synthesis process. **b** The selectivity of butenes and conversion of propene at 30 and 50 °C. **c** Conversion of CO from 100 to 400 °C with 1st–50th cycles. **a-c** Reproduced from Ref. [100] with permission. Copyright 2017 Springer Nature. **d** Schematic Illustration of the Synthesis of SA-Fe/CN. **e** HAADF-STEM image and corresponding EDX mapping of SA-Fe/CN, C (pink), N (green) and Fe (yellow). **f** AC HAADF-STEM image of SA-Fe/CN. **g** Benzene conversion catalyzed by the SA-Fe/CN, Fe nanoparticles/CN, CN, heme iron, phthalocyanine iron and ferric chloride, respectively. **d-g** Reproduced from Ref. [107] with permission. Copyright 2017 American Chemical Society

As a prototypical reaction in heterogeneous catalysis, CO oxidation has been extensively studied with variety of catalysts, especially Ru-, Au-, Pd- and Pt-based catalysts [98, 99]. However, the activity origin for the catalytic CO oxidation still hasn’t been fully explored. The recently developed SACs not only enable 100% atomic utilization efficiency, but also offer a new approach to further understand the mechanism at the atomic level. Yan and colleagues have decorated the isolated Pt atoms onto the mesoporous Al_2_O_3_ (Pt/m-Al_2_O_3_-O_2_) with varied loading contents [100]. The coordinately unsaturated Al^3+^ sites play a key role in anchoring Pt atoms via oxygen bridges. The as-synthesized hybrid Pt/m-Al_2_O_3_-O_2_ delivers excellent performance in the CO oxidation process (Fig. 6a–c). Encouragingly, the configuration of the isolated Pt centers, as well as the high catalytic performance, is well maintained in the long-term reaction period, which should be attributed to the mutual interactions between Pt and the support matrix.

Preferential oxidation of CO in H_2_-rich atmosphere is confirmed as the most direct and effective approach for removing CO and providing clean H_2_. Generally, oxide-stabilized Au catalysts are highly effective for CO oxidation but less active for H_2_ oxidation at low temperature. Zhang and co-workers have decorated the isolated Au centers onto the CeO_2_ support with the loading content of 0.05 wt%. (0.05Au_1_/CeO_2_) [37]. X-ray absorption fine structure (XAFS) studies clearly demonstrate that the Au_1_ atoms are located at the Ce vacancy sites. The developed catalyst delivers high CO conversion (> 99.5%) in the presence of rich H_2_ at elevated temperatures. The authors attribute the exceptional catalytic performance to the obviously reduced capability of the Au_1_ SACs to dissociate H_2_ in the catalytic process. Meng and colleagues have also tracked the origin of the superior CO oxidation performance of the isolated Pd atoms decorated graphene (PdGr) [101]. Combining DFT simulations and micro-kinetics modeling, the authors have confirmed that the positively charged PdGr can bind strongly with O_2_, acting as the reactive species to convert CO. The CO oxidation over the as-synthesized SAC mainly proceeds through revised Langmuir–Hinshelwood pathways, and the dissociation of the peroxide intermediate (O–O–C=O) is considered as the rate-limiting step.

#### Water–gas Shift (WGS) Reaction

WGS reaction is another crucial reaction in producing clean hydrogen and removing CO contamination for chemical processing, which also has been investigated on various supported catalysts. The related reaction mechanism is still under debate, especially for the real active sites in the catalytic process. Zhang and colleagues have anchored the isolated Ir atoms onto the FeO_x_ support (Ir_1_/FeO_*x*_) with rather low loading content (0.01 wt%) [38]. The developed catalyst exhibits impressive catalytic activity for the WGS reaction. The related reaction rate can reach up to 43.4 mol CO g_Ir_^−1^ h^−1^ at 300 °C. This value is nearly 1 order of magnitude higher than its nanoparticle or cluster counterparts, even higher than the most active Pt- or Au-based catalysts. The authors believe that the isolated Ir atoms can obviously improve the reducibility of the FeO_x_ support and thus generate amounts of oxygen vacancies, resulting in a greatly boosted catalytic activity for the WGS reaction.

Wang and coworkers have decorated isolated Pt atoms onto the FeO_x_ support (Pt-SAC) for WGS reaction. The developed catalyst delivers greatly improved specific activity (nearly 10 times) when compared with the nanoparticles [102]. With the Pt loading content of 0.05 wt%, Pt-SAC exhibits high CO conversion rate (~ 65%) at 300 °C. Experimental results show that Pt-SAC possesses the lowest activation energy for the WGS reaction. The isolated Pt atoms can accelerate the generation of oxygen vacancies on FeO_x_ and facilitate the dissociation of H_2_O to H_2_ and adsorbed O. The adsorbed O can interact with the weakly adsorbed CO over the isolated Pt sites and then realize the generation of CO_2_.

#### Methane Conversion

Direct conversion of natural gas into chemicals is an economical and eco-friendly approach. However, cleaving the first C-H bond in CH_4_ is still a great challenge for the high bonding strength in CH_4_ molecule. Song and colleagues have decorated isolated chromium atoms on TiO_2_ support for direct methane oxidation [103]. The optimized catalyst exhibits an impressive yield of 57.9 mol/mol_Cr_ and high selectivity (93%) at 50 °C for C_1_ oxygenated products. These values are among the highest values of most-recently reported catalysts. The quite good catalytic performance can be ascribed to the synergistic effect between isolated Cr atoms and TiO_2_ support. As proposed by the authors, the methane is firstly activated into the methyl radical, which is then converted into CH_3_OH. The generated CH_3_OH is further oxidized to HOCH_2_OOH and HCOOH. He and co-worker have further developed a bi-metallic oxide cluster anions of PtAl_2_O_4_^−^ to transform methane into formaldehyde with high selectivity under thermal collision conditions [104]. Mechanism investigations reveal that platinum atom rather than oxygen radical is the active species for CH_4_ activation at the beginning of the reaction. The Al_2_O_4_^−^ support also plays a crucial role in the late stage of the reaction.

As one typical example, isolated iron atoms confined in the silica lattice have been reported with high catalytic selectivity for the nonoxidative conversion of methane to ethylene, aromatics and hydrogen [105]. During the reaction, CH_4_ can be activated on the isolated Fe sites to initially generate methyl radicals, followed by a series of gas-phase reactions. Wang and his colleagues have also synthesized the nanoceria-supported atomic Pt catalysts (Pt_1_@CeO_2_) and confirmed their advanced catalytic performance for the nonoxidative conversion of methane to C_2_ products (acetylene, ethane and ethylene) [106]. Mechanism investigations reveal that the isolated Pt atoms may be capable of stabilizing C_2_ adsorbates formed from the catalytic coupling of two dehydrogenated C_1_ adsorbates on the single Pt sites.

#### Hydroxylation of Benzene

Hydroxylation of benzene to phenol is a key reaction in chemical industry. Pursuing the generation of phenol with low energy input, high yield and selectivity is always the goal of scientific researchers. Thus, developing high-efficiency and durable catalysts for the selective hydroxylation of benzene should be a crucial research topic. Li and colleagues have successfully confined the isolated Fe atoms onto the inner wall of a hollow carbon nanotube by employing a core–shell strategy (Fig. 6d–f) [107]. Impressive benzene conversion rate (45%) and phenol selectivity (94%) have been realized over the Fe-based SACs. These values are much higher than those of Fe nanoparticle-based catalysts (Fig. 6g). DFT simulation confirms that the exceptional activity should originate from the easier generation of activated oxygen species over the isolated Fe centers.

Wu and co-workers have also developed a cation-exchange strategy for decorating isolated metal atoms onto the edge-rich nitrogen and sulfur co-doped graphene (SAC/S–N) [108]. They find that the Cu SAC/S–N is the most suitable catalyst for benzene oxidation reaction among all the synthesized SACs (such as Pt, Au, and Ag), producing the high benzene conversion of 42.3% and a phenol selectivity of 93.4% at room temperature in 24 h. DFT simulations suggest that edge-rich S, N co-doped support could lower the reaction energy barrier of Cu sites, which greatly accelerates the thermodynamical process of the hydroxylation of benzene.

### Photocatalytic Reactions

Photocatalytic reactions driven by solar energy play an imperative role in conquering the energy crisis and environmental pollution. Many photocatalytic reaction systems have been established in the past decades. However, the catalytic performances are still far from satisfactory because of the rapid electron–hole recombination and low active surface areas. SACs have received much attention in photocatalysis due to their maximal atomic utilization and fascinating strengths in enhancing light-harvesting, charge transfer dynamics and surface reactions [92, 109–112].

#### Photocatalytic HER

In 2014, Yang et al. have successfully deposited isolated metal atoms (Pd, Ru, Pt, and Rh) onto the TiO_2_ support as a model photocatalytic reaction system [113]. Those well-synthesized SACs display superior photocatalytic hydrogen evolution activity when comparing to the counterpart metal clusters on TiO_2_. Experimental results and DFT simulation suggest that the impressive photocatalytic hydrogen evolution performance of TiO_2_-supported isolated Pt atoms can be ascribed to the decreased H* adsorption energy relative to that of metallic Pt, which is closer to the optimum thermodynamically.

**Fig. 7:**
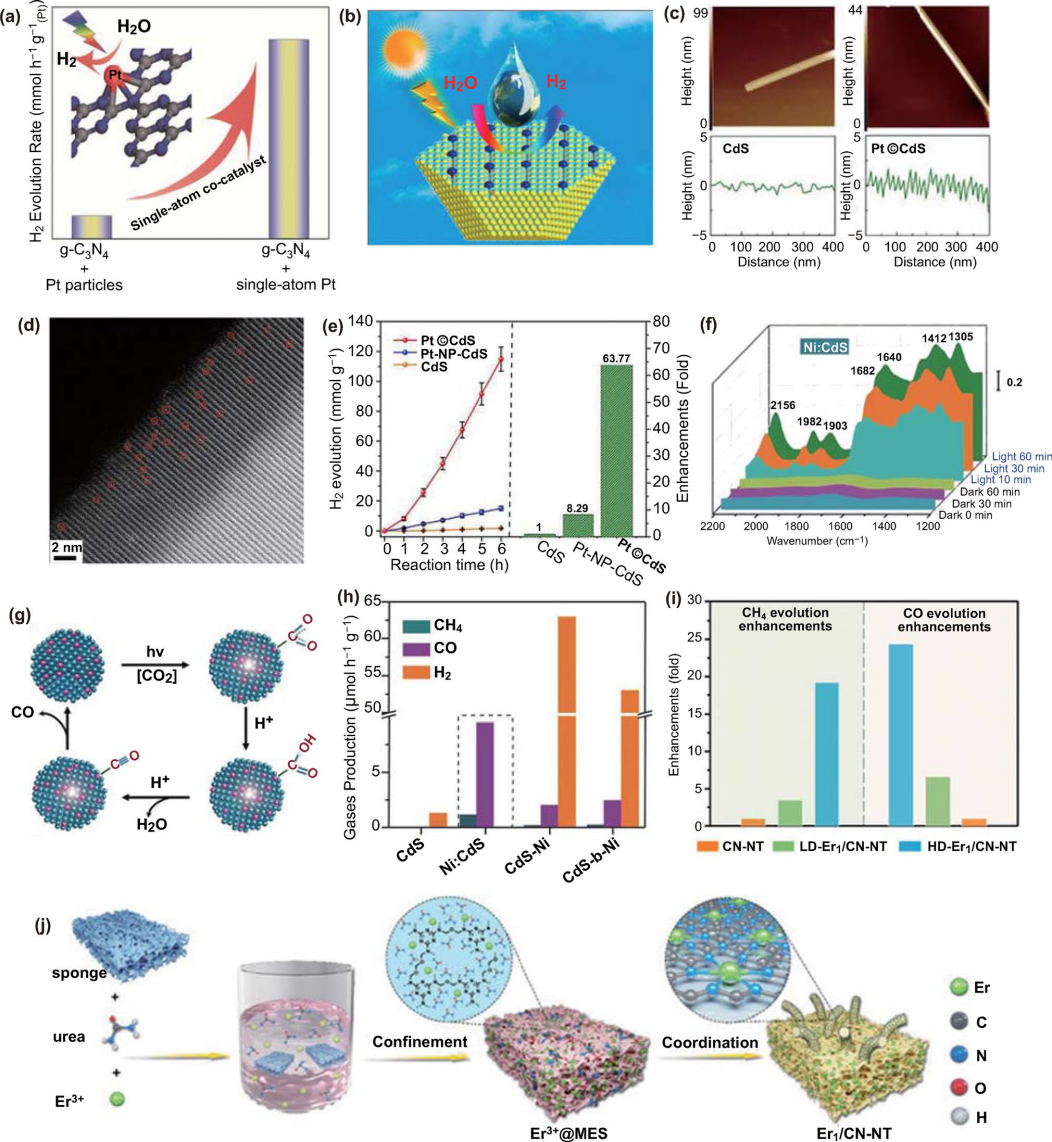
**a** Pt-CN for photocatalytic H_2_ evolution. Reproduced from Ref. [114] with permission. Copyright 2016 WILEY–VCH Verlag GmbH & Co. KGaA, Weinheim. **b** The geometric models of isolated platinum atoms decoration on the CdS surface with terraces for photocatalytic hydrogen evolution. **c** AFM maps and line scans for the CdS and Pt©CdS samples. **d** HAADF-STEM image in a spherical-aberration-corrected TEM for Pt©CdS. **e** Time dependent hydrogen evolution and enhancement of evolution over Pt©CdS (0.27 wt% Pt loading), Pt-NP-CdS (0.25 wt% Pt loading) and CdS. **b-e** Reproduced from Ref. [115] with permission. Copyright 2017 Elsevier Ltd. **f** In situ DRIFTS spectra for the reaction of CO_2_ with H_2_O on Ni(0.26%):CdS QDs under irradiation. **g** Proposed mechanism for the photocatalytic CO_2_ reduction at Ni:CdS QDs. **h** Average production rates of CH_4_, CO, and H_2_ using various photocatalysts based on CdS QDs. **f–h** Reproduced from Ref. [116] with permission. Copyright 2018 Wiley–VCH Verlag GmbH & Co. KGaA, Weinheim. **i** Enhancement of product evolution over the LD-Er_1_/CN-NT, HD-Er_1_/CN-NT, and CN-NT catalysts. **j** Schematic process of the synthesis of single-atom Er_1_/CN-NT catalysts. **i–j** Reproduced from Ref. [118] with permission. Copyright 2020 Wiley–VCH Verlag GmbH & Co. KGaA, Weinheim

Xie and co-workers have decorated the isolated Pt atoms onto the g-C_3_N_4_ (Pt-CN) [114]. The prepared Pt-CN exhibits a prominently boosted activity for photocatalytic water splitting into hydrogen (Fig. 7a). The photo-excited electron transfer investigation suggests that the isolated Pt atoms can intrinsically change the surface trap states of g-C_3_N_4_, which should be the main reason for the dramatically boosted photocatalytic performance. Furthermore, Ye et al. have decorated the isolated Pt atoms onto the surface step of CdS nanowires (Pt©CdS) (Fig. 7b–e) [115]. Pt©CdS exhibits a greatly improved photocatalytic performance for hydrogen evolution, which is 7.69 times higher than that of Pt nanoparticles and 63.77 times as high as the bare CdS nanowires. DFT simulations further suggest that the impressive catalytic activity should be ascribed to the decoration of positively charged Pt atoms with partially vacant 5d orbitals. The existence of Pt atoms can greatly influence the charge density distribution and facilitate the transfer of the photo-generated electron.

#### ***Photocatalytic CO***_***2***_*** Reduction Reaction***

The solar-driven CO_2_ reduction offers a highly important route to mitigate atmosphere CO_2_ emissions and convert solar energy into value-added chemicals. Amidst the numerous photocatalytic CO_2_ reduction systems in the past decade, SACs have exhibited great potential in achieving practical applications.

To overcome the limitations of low selectivity and stability of the established catalysts for CO_2_ reduction, Xiong et al. have doped isolated Ni atoms into the CdS quantum dots (Ni:CdS QDs) [116]. The obtained catalyst affords nearly 100% selectivity for the CO_2_ reduction into CO and CH_4_, a TON of approximately 35 in terms of the Ni atoms and excellent durability for more than 60 h (Fig. 7f-h). The doped isolated Ni atoms can trap the photo-excited electrons at surface catalytic sites and suppress the H_2_ evolution, playing a key role in the photocatalytic process. Liu and colleagues have further introduced isolated Co atom into Bi_3_O_4_Br atomic layers [117]. The developed catalyst can deliver a high selectivity and activity in the light-driven CO_2_ reduction to CO, about 32 and 4 times higher than those of bulk Bi_3_O_4_Br and atomic layer Bi_3_O_4_Br, respectively. DFT simulation reveals that the isolated Co atoms in the Bi_3_O_4_Br can stabilize the COOH* intermediates and modulate the rate-limiting step, resulting in greatly accelerated reaction kinetics.

Wang and coworkers have also decorated isolated rare-earth single erbium (Er) atom onto the carbon nitride nanotubes (Er_1_/CN-NT) with varied loading contents (Fig. 7i-j) [118]. The newly developed Er_1_/CN-NT displays prominent photocatalytic CO_2_ reduction activity in a pure-water system. Systematic theoretical reaction mechanism simulations clearly reveal the favorable formation of gaseous CO than CH_4_ and unfavorable H_2_ production on the isolated rare-earth Er atoms in Er_1_/CN-NT.

### Electrocatalytic Reactions

#### Electrocatalytic HER

**Fig. 8:**
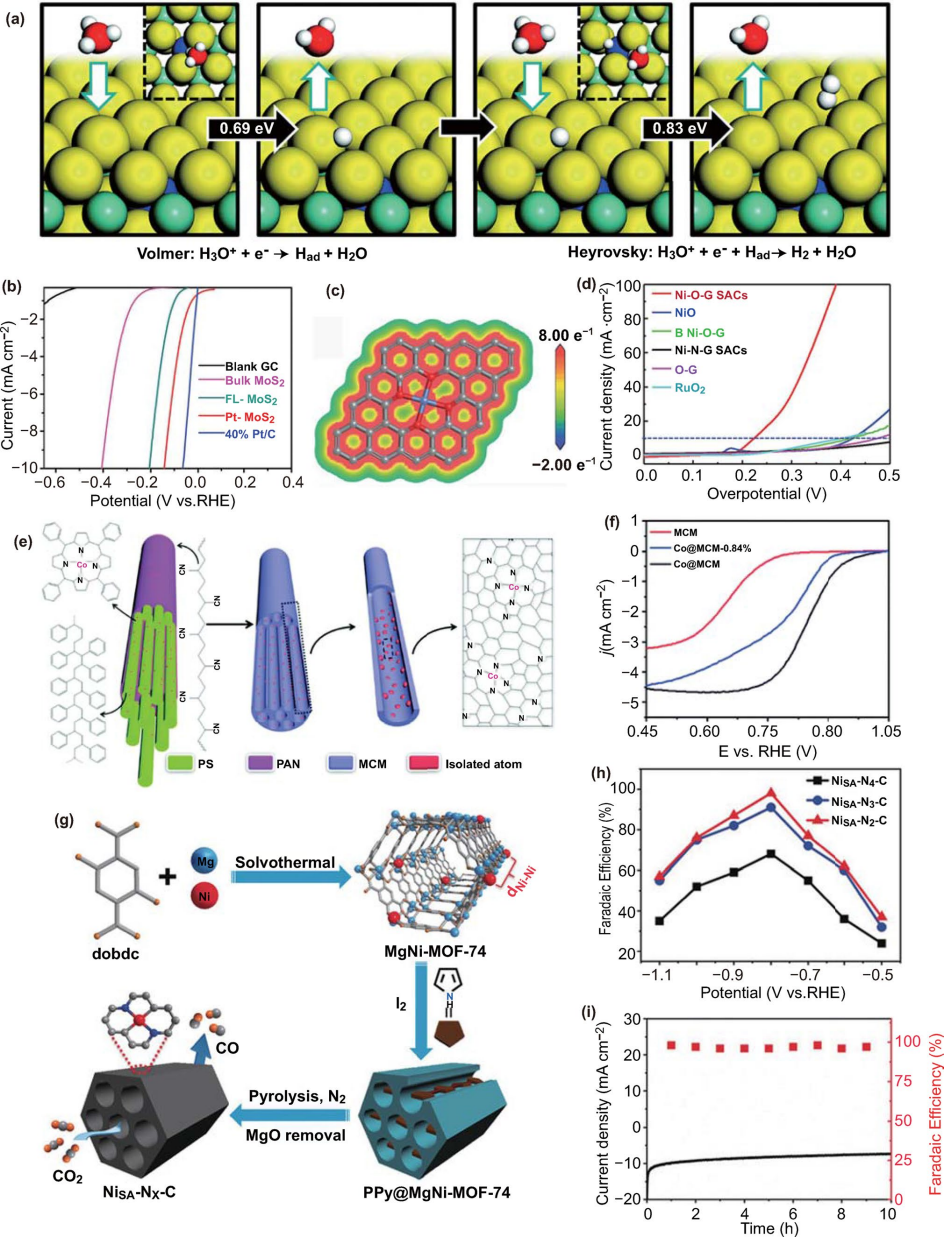
**a** HER process on a Pt-MoS_2_ catalyst. The top views are shown in the insets. The reaction barriers are shown in the black arrows. The green, yellow, blue, red and white balls represent Mo, S, Pt, O and H atoms, respectively. **b** HER polarization curves for Pt-MoS_2_ in comparison with blank GC electrode, bulk MoS_2_, FL-MoS_2_, and 40% Pt/C. **a–b** Reproduced from Ref. [126] with permission. Copyright 2015 The Royal Society of Chemistry. **c** Map of the DFT ESP surfaces of Ni–O–G SACs structure. Blue color indicates positive charges, and red color indicates negative charge. **d** The OER current curves of Ni–O–G SACs, NiO, B Ni–O–G, Ni–N–G SACs, O–G, and RuO_2_. **c–d** Reproduced from Ref. [135] with permission. Copyright 2020 The Authors. Published by WILEY–VCH Verlag GmbH & Co. KGaA, Weinheim. **e** Schematic illustration of the synthetic process of Co@MCM. **f** ORR LSV curves of MCM, Co@MCM-0.84% and Co@MCM in O_2_-saturated 0.1 M KOH solution. **e–f** Reproduced from Ref. [156] with permission. Copyright 2018 The Royal Society of Chemistry. **g** Illustration showing the host–guest cooperative protection strategy for the fabrication of Ni_SA_-N_x_-C catalysts for electrocatalytic CO_2_ reduction. **h** FEs of CO at different applied potentials. **i** Stability of Ni_SA_-N_x_-C at − 0.8 V during 10 h. **g–i** Reproduced from Ref. [161] with permission. Copyright 2019 Wiley–VCH Verlag GmbH & Co. KGaA, Weinheim

The electrochemical HER offers a clean and sustainable approach to produce H_2_ [119–124]. Pt-based catalysts have been regarded as the most active electrocatalysts to catalyze HER [125]. Bao and colleagues have decorated the isolated Pt atoms into MoS_2_ (Pt-MoS_2_) via a one-pot chemical synthetic approach [126]. The inert in-plane S atoms of MoS_2_ can be greatly activated via the decoration of isolated Pt atoms, which is confirmed by the greatly improved HER performance. According to the DFT calculations, the activation of in-plane MoS_2_ should be directly reflected from the greatly changed adsorption behavior of H atoms over S atoms by neighboring doped Pt atoms (Fig. 8a, b).

Remarkable efforts have also been contributed to the development of efficient non-noble electrocatalysts as Pt alternatives [68, 127–129]. Liu and coworkers have decorated the isolated Co atoms into the lattice of the distorted 1 T MoS_2_ nanosheets (SA Co-D 1 T MoS_2_), which exhibits quite high long-term stability and impressive activity comparable to the Pt-based catalysts [130]. DFT simulation suggests that the impressive catalytic performance should be ascribed to the greatly modified hydrogen binding over the S atoms after the Co decoration. Tour and coworkers have incorporated trace amounts of individual Co atoms on the nitrogen-doped graphene [131]. The identified Co atoms behaving as extraordinary active sites can minimize reaction kinetic barriers with a low onset potential of ~ 30 mV for hydrogen generation.

#### Electrocatalytic Oxygen Evolution Reaction (OER)

The high production of hydrogen through electrocatalytic water splitting is usually hindered by the kinetically sluggish OER. Therefore, highly efficient and robust SACs are urgently needed to enhance OER efficiency for minimizing the total overpotential for water splitting and other electronical applications [132–134]. Recently, Bao et al. have anchored individual high-valence nickel atoms on graphene-like carbon via oxygen sites (Ni–O-G SACs) [135]. The developed catalyst delivers impressive activity for OER. Low overpotential of 224 mV is needed to realize the current density of 10 mA cm^−2^. Its Tafel slope (42 mV dec^−1^) and turnover frequency (TOF) of oxygen production at 300 mV (1.44 S^−1^) have also been investigated and the obtained values are among the highest reported values for OER (Fig. 8c, d). The DFT simulations reveal that the highly oxidized Ni species in the configuration of Ni-O_4_(OH)_2_ can reduce the Gibbs-free energy and extraordinarily boost OER performance of Ni–O-G SACs.

Gu and coworkers have fabricated isolated Ir atoms on NiO with ultrahigh loading content (18 wt%) for OER [136]. The as-synthesized catalyst exhibits a 46 times increase in the OER current density compared with IrO_2_ at an overpotential of 260 mV. DFT calculations reveal that the substituted Ir atom can also improve the activity of surrounding Ni atoms. The increased catalytic sites and the cooperative interaction between isolated atoms and support are thereby responsible for the enhanced intrinsic activity.

#### Electrocatalytic ORR

ORR plays a crucial role in the energy storage and conversion systems such as fuel cells and some other renewable energy technologies [137–139]. So far, Pt and its alloys are typically regarded as the most efficient ORR catalysts [140–143]. Recently, Chou et al. have constructed a quasi-Pt-allotrope electrocatalyst (H-PtCo@Pt_1_N-C), composed of hollow Pt_3_Co alloy cores and isolated Pt atoms decorated N-doped carbon matrix as shell [144]. This unique nanoarchitecture can prevent the agglomeration of hollow Pt_3_Co alloy (H-PtCo) core and increase the ORR performance in virtue of the isolated Pt atoms. The H-PtCo@Pt_1_N-C catalyst shows an efficient ORR activity in various organic electrolytes. Chen and coworkers have developed an Ir-N–C single-atom catalyst (Ir-SAC) to mimic homogeneous iridium porphyrins for high-efficiency ORR [145]. The as-synthesized Ir-SAC delivers orders of magnitude higher ORR activity than iridium nanoparticles. DFT simulations suggest that the obtained impressive performance should be ascribed to the optimized adsorption energy of reaction intermediates over the four nitrogen atoms coordinated iridium sites.

Tremendous attention has also been contributed to the fabrication of nonprecious-metal based ORR catalysts [146–154]. Bao and coworkers have confined isolated iron atoms via N atoms to form the FeN_4_ structure in the carbon matrix [155]. The developed catalyst delivers an impressive ORR activity, nearly comparable to the commercial 40% Pt/C catalyst. DFT simulations demonstrate that the nice ORR performance should be ascribed to the isolated Fe centers in the unsaturated configuration and high loading content. Lou and coworkers have decorated isolated cobalt atoms into a multichannel carbon matrix (Co@MCM) in the configuration of CoN_4_ (Fig. 8e) [156]. The unsaturated Co centers as well as the highly porous and conductive multichannel carbon substrate endow the catalyst with quite good ORR performance. DFT simulations verify that the barrier for the rate-determining step of O_2_* reduction can be greatly reduced by the penetration of isolated Co sites (Fig. 8f).

#### ***Electrocatalytic CO***_***2***_*** Reduction Reaction***

The electrocatalytic CO_2_ reduction into value-added products is an effective strategy to mitigate the energy crisis and related environmental issues [157, 158]. At present, the electrocatalytic CO_2_ reduction is faced with the large overpotential and a more complex reaction pathway than the above types of reactions due to the participation of various intermediates [159]. As a result, rational design of highly active and selective catalysts is important to regulate the CO_2_ reduction performance. Chen and colleagues have decorated the isolated Pd atoms into the nitrogen-doped carbon support for efficient electrocatalytic CO_2_ reduction [160]. This Pd SAC exhibits significantly improved CO_2_ reduction performance by avoiding the formation of H_2_. Further DFT calculation suggests that the Pd-N_4_ site should be the real active sites for the CO evolution. The adsorbed CO_2_ intermediate can be stabilized in the Pd-N_4_ sites and thus realize the electrocatalytic CO_2_ reduction at low overpotential.

Jiang et al. have introduced the isolated Ni atoms with controlled Ni–N coordination number into the N-doped carbon (Fig. 8g) [161]. Significantly, Ni_SA_-N_2_-C featuring two nitrogen atoms coordinated Ni sites affords the best CO Faradaic efficiency (98%) and turnover frequency (1622 h^−1^) among all the prepared Ni SACs in electrocatalytic CO_2_ reduction reaction (Fig. 8h, i). Theoretical calculations reveal that the unique electronic structure in Ni_SA_-N_2_-C enables a facile formation of COOH* intermediate and weak binding of CO, thus giving rise to its superior performance. Xie and coworkers have also loaded the isolated Mo atoms on the ultrathin N doped graphene to promote the intrinsic catalytic activity of the substrate [162]. The prepared Mo SAC exhibits an improved C_1_ product selectivity and far better formate production rate with the aid of 4 mol% ionic liquid than N doped graphene. DFT calculations reveal that the isolated Mo atoms can facilitate the preferred CO_2_ adsorption and the rate-determined reaction process, promoting the intrinsic electrocatalytic hydrogenation activity of N doped graphene.

## Conclusions and Outlook

Significant breakthroughs for the development of SACs have been achieved in the past five years. It is very encouraging that we have developed various strategies for tailoring the local electronic structure and coordination environment of the isolated reactive sites. In the current review, we summarize and discuss the recent achievements in engineering the coordination sphere to explore the intrinsic activity of the SACs in heterogeneous catalysis. Our profound understanding of the SACs has been rapidly established in the terms of catalyst synthesis, catalytic application and mechanism exploration. Although it has been validated that SACs, as a prospective research frontier, present the ultra-most atomic utilization efficiency, successful paradigms for the practical application are still limited. Some foreseeable challenges and critical issues are urgent to be addressed to foster the rational design of more efficient SACs. Besides, possible solutions for addressing the existed challenges are also elaborated as below:

(I) Notwithstanding great progress has been realized in preparing the SACs, the synthesis of SACs with high loading content of isolated metal atoms is still an open question. Therefore, in-depth comprehending the synthetic processes of SACs and exploiting feasible strategies are vital to improve the density of active sites in SACs and implement high-performance catalytic reactions for desirable catalytic systems. Several approaches should be considered in the rational design of SACs with high loading density. Increasing the support area and introducing the defects and vacancies are beneficial for increasing the loading contents of the isolated atoms.
(II) As many catalytic processes are complicated with multi steps, the isolated active site may fail to satisfy the activation of all intermediates. To conquer this problem, developing multi-metal sites at atomic level is quite important. The recently developed atom by atom strategy for constructing the atomically precise metal clusters holds great promise for bridging the SACs and particle-based catalysts. Moreover, different metal species can produce varied activation effects in the catalytic process. The active centers can be generally modulated by the incorporation of varied metal elements. They may interact with each other and work in synergy to facilitate the catalytic kinetics for the particular catalysis. Research along this direction will offer new comprehension on the structure–activity correlations. The achievements would certainly benefit the whole heterogeneous catalysis community.
(III) The design of SACs at atomic level will generally challenge the limits of characterization. At present, the structural nature of SACs is mainly identified by the combination of XAFS and high-resolution electron microscopy techniques, which fail to provide the most accurate geometric and electronic structure of the reactive centers. For example, it is hard to tell the difference between the M-C and M–N interactions since the scattering contributions of those coordinated atoms are nearly overlapped. Thus, developing atomically resolved electron microscopy techniques to distinguish different heavy metal as well as light elements is a continuous demand.
(IV) There is still a gap in the comprehension of catalytic kinetics and mechanism for the particular reaction types. With the help of *operando* characterization techniques, including the means of spectroscopic/microscopic/imaging techniques, the structural evolution can be well tracked, which contributes to understanding the reaction mechanism of SACs under practical operating conditions. Thus, more attention should especially concern the *operando* characterizations to monitor the structure evolution of reactive centers as well as their interaction with main intermediates and final products. The obtained results can feedback clues about catalytic pathways and further provide a new perspective to design versatile SACs.
(V) Thanks to the uniformly distributed active sites, it is feasible to perform a theory-guided reaction simulation and thus provide us a platform for further understanding the catalytic mechanism. The progress in the big-data provides us additional assistants for simulating the binding strengths between active centers and reactants/intermediates/products, the reaction pathways, as well as the reaction barriers of some pivotal steps involved in the reaction process. The achieved structure–activity relationships can predict the catalytic performances for screening materials and finally establish an elegant platform for designing new catalysts for energy related applications.
(VI) Directly measuring the intrinsic catalytic activity of a single atom in real electrochemical environments is still challenging. Typically, most reported SACs are measured by depositing thin layers of catalyst on carbon or some other supports. The catalytic activity of the entire electrode is measured, and contribution from individual atoms is inferred rather than directly measured. Recently, microelectrochemical measurements have been becoming a mature technique to study the intrinsic activity of catalyst at atomistic resolution. Taking advantaging of the imaging of single atom at specific support (e.g. carbon, MoS_2_) by advanced microscopic imaging characterization, the intrinsic activity of single atom can be measured in microelectrochemical cells.
